# Efficient Transmission of Subthreshold Signals in Complex Networks of Spiking Neurons

**DOI:** 10.1371/journal.pone.0121156

**Published:** 2015-03-23

**Authors:** Joaquin J. Torres, Irene Elices, J. Marro

**Affiliations:** Department of Electromagnetism and Physics of the Matter, University of Granada, Granada, Spain; Universiteit Gent, BELGIUM

## Abstract

We investigate the efficient transmission and processing of weak, subthreshold signals in a realistic neural medium in the presence of different levels of the underlying noise. Assuming Hebbian weights for maximal synaptic conductances—that naturally balances the network with excitatory and inhibitory synapses—and considering short-term synaptic plasticity affecting such conductances, we found different dynamic phases in the system. This includes a memory phase where population of neurons remain synchronized, an oscillatory phase where transitions between different synchronized populations of neurons appears and an asynchronous or noisy phase. When a weak stimulus input is applied to each neuron, increasing the level of noise in the medium we found an efficient transmission of such stimuli around the transition and critical points separating different phases for well-defined different levels of stochasticity in the system. We proved that this intriguing phenomenon is quite robust, as it occurs in different situations including several types of synaptic plasticity, different type and number of stored patterns and diverse network topologies, namely, diluted networks and complex topologies such as scale-free and small-world networks. We conclude that the robustness of the phenomenon in different realistic scenarios, including spiking neurons, short-term synaptic plasticity and complex networks topologies, make very likely that it could also occur in actual neural systems as recent psycho-physical experiments suggest.

## Introduction

Many physical systems present ambient and intrinsic fluctuations that often are ignored in theoretical studies to obtain simple mean-field analytical approaches. Nevertheless, these fluctuations may play a fundamental role in natural systems. For instance, they may optimize signals propagation by turning the medium into an excitable one—e.g., the case of ionic channel stochasticity in neurons that can affect the first spike latency [1, 2] or enhance signal propagation through different neuronal layers [3] –, originate order at macroscopic and mesoscopic levels [4, 5] or induce coherence between the intrinsic dynamics of a system and some weak stimuli it receives, a phenomenon known as *stochastic resonance* (SR) (see for instance [6] for a review). Precisely, this intriguing phenomenon has attracted the interest of the computational neuroscience community for its possible implications in the complex processing of information in the brain [7–11], or as a way to control specific brain states [12]. In fact, most neural systems naturally include the main factors involved in stochastic resonance, namely, different sources of intrinsic and external noise and complex nonlinear dynamic processes associated, for instance, to neuron excitability and information transmission through the synapses. Thus, several experimental, theoretical and numerical studies concerning the efficient transmission of weak signals in noisy neural systems have been reported recently [7–11, 13–17].

Particularly interesting are recent works that show that, differently to what happen in traditional SR phenomena, noisy neural systems can optimally process relevant information at more than one level of the ambient noise [15, 17]. However, it is not clear yet what are the main factors responsible for this new phenomenology. It has been reported, for instance, that this new phenomenon can appear in single perceptrons with dynamic synapses [15] as a consequence of the complex interplay between dynamic synapses and adaptive threshold mechanisms affecting neuron excitability. Also, similar phenomena has been reported in binary networks with scale-free topologies [13], so that these resonances may emerge as a consequence of topological disorder. Finally, very recently, it has been reported that optimal processing of weak relevant signals at different levels of the underlying noise also occurs in *auto-associative* networks of binary neurons with dynamic synapses [17]. Most of these studies, however, consider very simple models at the neuron, network and synapse levels. This makes difficult to extrapolate their results and conclusions to actual neural systems.

Here, we present a full computational study of how weak relevant subthreshold signals can be processed by neural systems in a more realistic scenario, that is, a complex auto-associative network of spiking neurons with dynamic synapses. We consider a network of *N* *integrate and fire* neurons, and assume a long-term synaptic plasticity mechanism, due to Hebbian learning, affecting the synapses connecting the neurons. In order to increase the biological relevance of our study we also consider different types of *short-term synaptic plasticity*, such as the so called *short-term depression* and *short-term facilitation*. These mechanisms introduce synaptic changes at short time scales, as it is expected to occur due to the existence of a limited amount of neurotransmitters at each synapse that can be released after some presynaptic stimulus, which needs some time to recover after this stimulus.

In addition, to mimic any source of intrinsic or external ambient noise that can affect neuron dynamics and its excitability, we add to each neuron dynamics a source of uncorrelated Gaussian noise together with a weak stimulus. In this way, by controlling the intensity of noise we can monitor the levels of the noise at which the processing of the weak stimulus by the system is more efficient. For simplicity, we assume in most of the cases reported here a form of sinusoidal signal for the stimulus, which simulates some relevant information to be processed by neurons, but other type of more realistic signals can also be considered with the same results (see Results section).

To see how efficient this weak stimulus in each neuron is processed by the system, one can compare, for instance, the coherence in time between the mean network activity and the stimulus by means some information transfer measurement as a function of the noise intensity. In general for low noise, the activity of the system cannot correlate with the stimulus due to its small amplitude. If the intensity of noise is increased sufficiently, then the noise is able to enhance the stimulus temporal features in such a way that the network activity can start to correlated with it and a peak of information transfer will appear. Nevertheless, if the noise intensity is too high, the network activity will be dominated by the noise preventing the input stimulus from being detected by the system.

On the other hand, activity dependent synaptic mechanisms, such as short-term depression and short-term facilitation, may be highly relevant in signal detection in noisy environments and can play a main role, for instance, in SR [15, 17]. These synaptic mechanisms may modify the postsynaptic neural response in a nontrivial way. Synapses can present short-term depression when the amount of neurotransmitters that are available for release whenever an action potential arrives is limited, and consequently, the synapse may not have time to recover them if the frequency of arriving spikes is too high. On the contrary, short-term facilitation is determined by the excess of calcium ions in the presynaptic terminal which can increase the postsynaptic response under repetitive stimulation. Both synaptic processes could interact with noise and some neuron excitability and adaptive mechanisms to induce a strong influence during the processing of relevant signals or stimulus in the brain. In particular, it has been recently reported in single perceptrons and in network of binary neurons that the complex interplay among these synaptic mechanisms allow for efficient detection of weak signals at different levels of the underlying noise [15, 17] and maintain coherence for a wide range of the intensity of such noise [8].

In this work, we demonstrate that these intriguing emergent phenomena appear also during the processing of weak subthreshold signals in more realistic neural media and in many different conditions. Therefore, it is highly likely that also they may appear in the actual neural systems, where different types of signals and stimulus are continuously processing in the presence of different sources of intrinsic and external noise. Moreover, the fact that the processing of weak subthreshold signals occurs at well defined different levels of noise—normally one relatively low and the other relatively high—can have strong implications concerning how the signal features are being processed. This can be clearly depicted in the case of more realistic Poissonian signals (see Results section)

To demonstrate the robustness of our findings, we performed a complete analysis of the emergent phenomena changing many variables in our system. This confirms that the same interesting phenomena emerges in all these situations, including, for instance the case in which the number of neurons in the network and the number of stored patterns is increased. In addition, the phenomenon of interest also remains for non-symmetric stored patterns provided that there is a phase of transitions between a high activity state (up state) and a low activity state (down state) in the network activity. Including short-time synaptic facilitation at the synapses, competing with synaptic depression, causes also intriguing features. This includes a dependency with the level of facilitation, of the level of noise at which the subthreshold signals are processed and detected and an enhancing of the detection quality for large facilitation. Finally, we checked the robustness of our finding for more realistic network topologies, such as diluted networks, and complex scale-free and small-world topologies confirming that phenomenon is robust also in these cases.

## Materials and Methods

The system under study consists of a spiking network of *N* integrate and fire neurons interconnected each other. The membrane potential of the *i* − *th* neuron then follows the dynamics

$$\begin{array}{c} \tau_{m}\frac{dV_{i}\left(t\right)}{dt}=-V_{i}\left(t\right)+R_{m}I_{i}\left(t\right)\;0<V_{i}\left(t\right)<V_{th}, \end{array}$$ (1)

where *τ*_*m*_ is the cell membrane time constant, *R*_*m*_ is the membrane resistance and *V*_*th*_ is a voltage threshold for neuron firing. Thus, when the input current *I*_*i*_(*t*) is such that depolarizes the membrane potential until it reaches *V*_*th*_ = 10*mV* an action potential is generated. Then, the membrane potential is reset to its resting value—that for simplicity we assume here to be zero—during a *refractory period* of *τ*_*ref*_ = 5 *ms*. We can assign binary values *s*_*i*_ = 1, 0 to the state of the neurons, depending if they have their membrane voltage above or below the voltage firing threshold *V*_*th*_. Furthermore, we assume that synapses between neurons are *dynamic* and described by the Tsodyks-Markram model introduced in [18].

Within this framework, we consider that the total input current *I*_*i*_(*t*) in the Equation (1) has four components, that is, $I_{i}(t)=I_{0}+I_{i}^{ext}+I_{i}^{syn}+D\zeta (t)$ where *I*_0_ is a constant input current. The second term $I_{i}^{ext}$ represents an external input weak signal which encodes relevant information and for simplicity we assume to be sinusoidal, that is

$$\begin{array}{c} I_{i}^{ext}=d_{s}\sin\left(2\pi f_{s}t\right), \end{array}$$ (2)

with frequency *f*_*s*_ and a small amplitude *d*_*s*_. The fourth component of *I*_*i*_(*t*) is a noisy term that tries to mimic different sources of intrinsic or external current fluctuations, where *ζ*(*t*) is a Gaussian white noise of zero mean and variance *σ* = 1, and *D* is the noise intensity. Finally, the third component $I_{i}^{syn}$ is the sum of all synaptic currents generated at neuron *i* from the arrival of presynaptic spikes on its neighbors. Following the model of dynamic synapses in [18], we describe the state of a given synapse *j* by variables *y*_*j*_(*t*), *z*_*j*_(*t*) and *x*_*j*_(*t*) representing, respectively, the fraction of *neurotransmitters* in active, inactive and recovering states. Within this framework, active neurotransmitters *y*_*j*_(*t*) are the responsible for the generation of the postsynaptic response after the incoming presynaptic spikes, and become inactive after a typical time *τ*_*in*_ ∼ 2 − 3*ms*. On the other hand, inactive neurotransmitters can recover during a typical time *τ*_*rec*_ which is order of a half second for typical pyramidal neurons [18], a fact that induces *short-term synaptic depression*. Recovered neurotransmitters become immediately active with some probability 𝒰—the so called release probability—every time a presynaptic spike arrives to the synapses. In actual synapses, 𝒰 can increases in time with a typical time constant *τ*_*fac*_—due to some cellular biophysical processes associated to the influx of calcium ions after the arrival of presynaptic spikes—which induces the so called *short-term synaptic facilitation*.

The synaptic current generated at each synapse then is normally assumed to be proportional to the fraction of active neurotransmitters, namely *y*_*j*_(*t*), so the total synaptic current generated in a postsynaptic neuron *i* is:

$$\begin{array}{c} I_{i}^{syn}=\sum_{j}^{N}𝒜\;y_{j}\left(t\right)\;J_{ij}\epsilon_{ij}. \end{array}$$ (3)

Here 𝒜 is the maximum synaptic current that can be generated at each synapses, *ε*_*ij*_ is the adjacency matrix that accounts for the connectivity matrix in the neural medium, and *J*_*ij*_ are fixed parameters modulating the synaptic current which can be related, for instance, with maximal synaptic conductance modifications due to a slow learning process. In this way, one can choose these *synaptic weights* *J*_*ij*_ following, for instance, a Hebbian learning prescription, namely:

$$\begin{array}{c} J_{ij}=\frac{\kappa }{\left\langle k\rangle a(1-a\right)}\sum_{\mu }^{P}\left(\xi_{i}^{\mu }-a\right)\left(\xi_{j}^{\mu }-a\right),\;J_{ij}=J_{ji},\;J_{ii}=0. \end{array}$$ (4)

Here, *J*_*ij*_ contains information from a set of *P* patterns of neural activity, namely $\left\{\xi_{i}^{\mu }=0,1\right\}$, with *μ* = 1, …, *P* and *i* = 1, …, *N* that are assumed to have been previously stored or memorized by the system during the learning process. Here $\xi_{i}^{\mu }$ denotes the firing (with membrane voltage above *V*_*th*_) or silent (with *V*_*m*_ below *V*_*th*_) state of a given neuron in the pattern *μ*. The parameter *a* measures the excess of firing over silent neurons in these learned patterns, or more precisely $a={\langle \xi_{i}^{\mu }\rangle }_{i,\mu }$. Since ∣*J*_*ij*_∣ can be in general very small and it is multiplying the single synapse currents, we have considered in (4) an amplification factor *κ* = 2000 to ensure a minimum significant effect of the resulting synaptic current (3) in the excitability of the postsynaptic neuron. Moreover, we also choose a mean node degree factor ⟨*k*⟩, instead of *N* in the denominator of *J*_*ij*_ which is more appropriate since it gives a similar mean synaptic current per neuron for all type of network topologies considered in this study, including fully connected networks, diluted networks and complex networks such as scale-free and the classical Watts-Strogatz small-world networks [19].

Following standard techniques from binary attractor neural networks, we can measure the degree of similarity between a state of the network and a certain stored activity pattern by means of an *overlap* function *m* ^*μ*^(*t*) defined as:

$$\begin{array}{c} m^{\mu }\left(t\right)=\frac{1}{aN(1-a)}\sum_{i=1}^{N}\left(\xi_{i}^{\mu }-a\right)s_{i}\left(t\right), \end{array}$$ (5)

as well as describe the activity of the system through the mean firing rate:

$$\begin{array}{c} \nu \left(t\right)=\frac{1}{N}\sum_{i}s_{i}\left(t\right). \end{array}$$ (6)

In order to visualize if our system is able to respond efficiently to some input weak stimulus, it is useful to quantify the intensity of the correlation, during a time window *T*, between the weak input signal and the network activity by computing, for instance, the Fourier coefficient at a given frequency *f*, of the network mean firing rate, that is,

$$\begin{array}{c} C_{f}=\lim_{T\to \infty }\frac{1}{T}\int_{0}^{T}\nu \left(t\right)e^{ift}dt. \end{array}$$ (7)

The relevant correlation, denoted *C*(*D*) in the following, it then is defined as the value of

$$\begin{array}{c} C\left(D\right)\equiv \frac{|C_{f_{s}}{|}^{2}}{d_{s}^{2}}, \end{array}$$ (8)

that is, the ration between the power spectrum computed at the frequency of the input signal *f*_*s*_ and the amplitude of this weak signal.

## Results

### The effect of short-term synaptic depression

**Fig 1:**
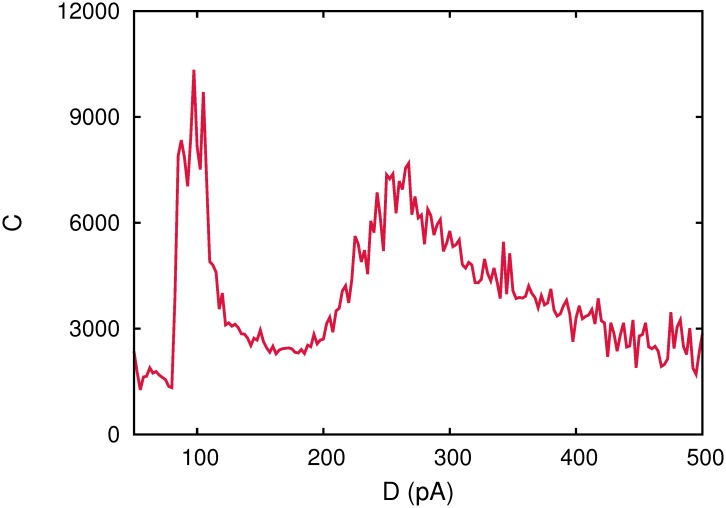
Correlation function *C*(*D*) for a single realization of a fully connected network of *N* = 1600 spiking neurons with pure depressing synapses with *τ*_*rec*_ = 300 *ms* and one stored pattern *P* = 1. Other parameters were *a* = 0.5, *κ* = 2000, *I*_0_ = 100 *pA*, *d*_*s*_ = 20 *pA*, *f*_*s*_ = 1.5 *Hz*, 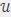 = 0.5, 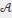 = 45 *pA*, *τ*_*in*_ = 3 *ms*.

As it was stated above, it is important to investigate the mechanisms involved in the processing of different stimulus by a neural system, in the presence of noise. This would determine the conditions of ambient or intrinsic noise at which the transmission of information can be more efficient mainly, when the relevant information of the stimulus is encoded in weak signals. This is particularly important in a complex neural system as it is the brain, where certain brain areas have to respond adequately to some signals, for instance, arriving from other specific brain areas or the senses, within a background of noisy activity. Following this aim, we have first studied how efficient is the processing of noisy weak signals in a network of *N* spiking neurons, when it stores a single pattern of neural activity, and where the synapses among neurons present short-term synaptic depression. Our study reveals the relevant signals can be processed by the systems at more than one level of the underlying noise, as it is depicted in Fig. 1. More precisely, the correlation measure *C*(*D*) presents, two well defined maxima, one at relatively low noise *D*_1_ = 97.5 *pA* and the second at relatively large noise intensity *D*_2_ = 265 *pA*. Model parameter values are indicated in the caption of the Fig. 1.

**Fig 2:**
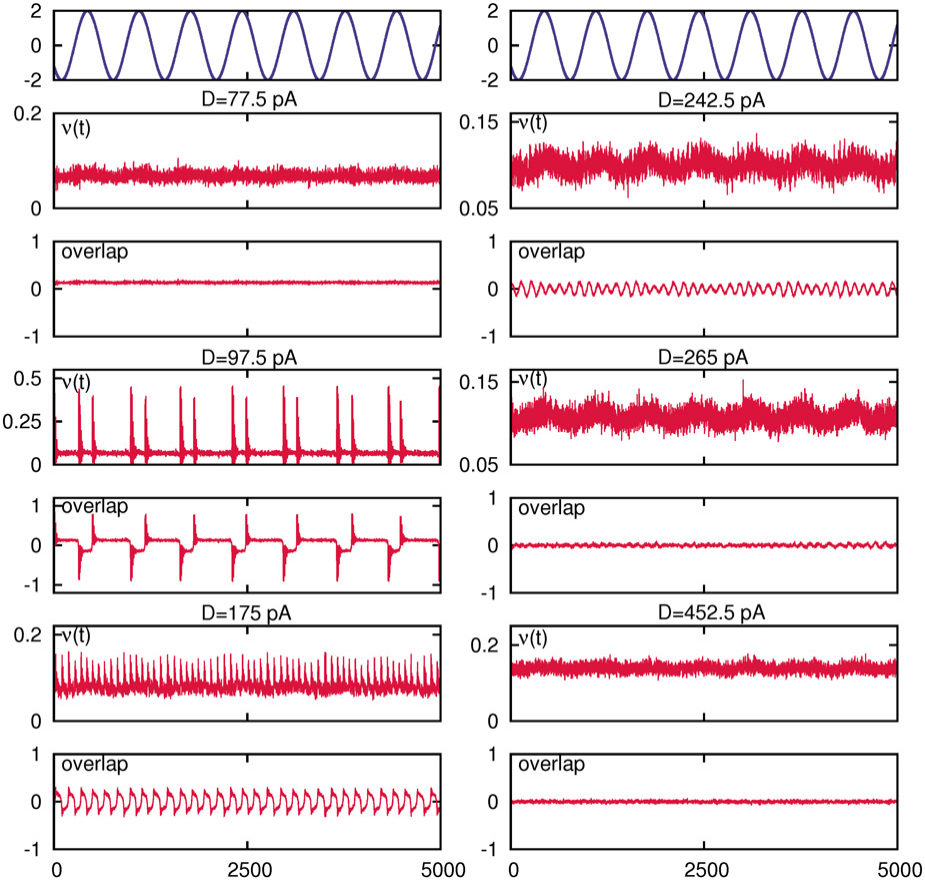
Temporal behavior of the mean firing rate and the overlap function for five different values of the noise intensity *D* along the curve *C*(*D*) depicted in Fig. 1. This shows a tendency to the coherence between network activity and the weak stimulus around two values of the noisy intensity, namely *D*_1_ = 97.5 *pA* and *D*_2_ = 265 *pA*. The weak sinusoidal stimulus is also shown on the top panels.

**Fig 3:**
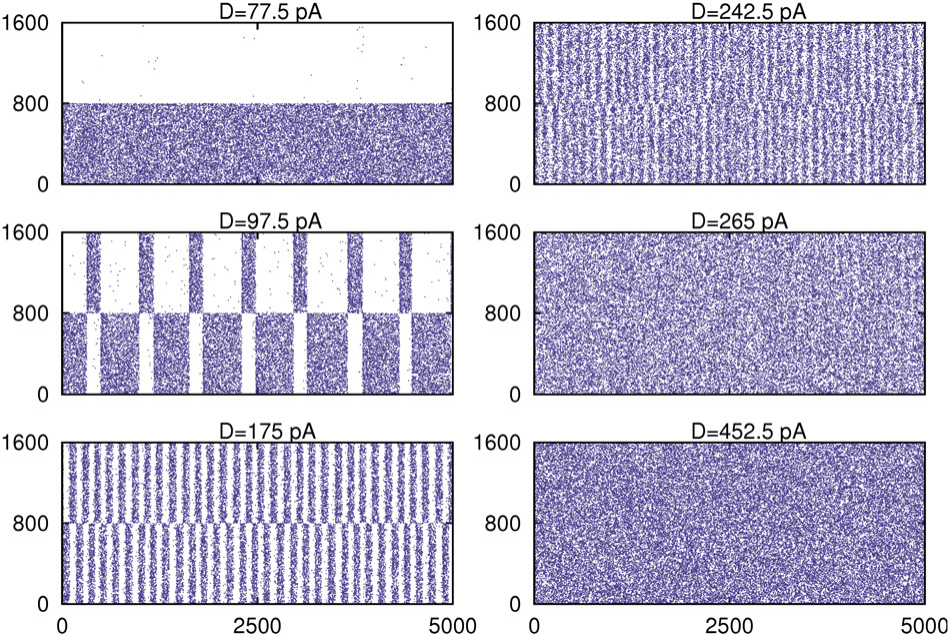
Raster plots of the network activity for different values of the noise intensity *D* for the network realization with the correlation *C*(*D*) depicted in Fig. 1. The stored pattern in such that for *i* = 0, …, 799 *s*_*i*_ = 1, that is neurons are active, and for *i* = 800, …, 1600 *s*_*i*_ = 0 that is, these neurons are silent in the pattern. At the first maximum of *C*(*D*) that occurs around *D*_1_ = 97.5*pA* there are oscillations of the network activity around the stored pattern and its anti-pattern which are correlated with the weak stimulus. At a second critical level noise *D*_2_ = 265*pA* a transition between the above oscillatory phase and a disordered phase appears, and a second maximum of *C*(*D*) emerges where a very noisy mean activity in the network also is correlated with the weak stimulus. All depicted panels corresponds to the same cases shown in Fig. 2.

A full description of collective behavior of the network, by means of the temporal evolution of the mean firing rate and the overlap function compared with the weak sinusoidal input, and for increasing values of the noise parameter *D* along the curve *C*(*D*), is depicted in Fig. 2. Moreover, raster plots of the network activity, for the same cases shown in Fig. 2, are presented in Fig. 3.

Both figures show (more clearly illustrated in Fig. 3), that for relatively low noise the system is able to recall the stored pattern, which becomes an attractor of the system dynamics. The system, therefore, shows the associative memory property. When noise intensity is increased to some given value *D*_1_ (around 97.5 *pA* in this figure), the dynamic regime of the system changes *sharply* to an oscillatory phase where the network activity periodically switches between a pattern and anti-pattern configurations. Around this phase-transition point *D*_1_, these oscillations start to be driven by the weak signal which causes the first appearing maxima in *C*(*D*). This periodically switching behavior correlated with the weak signal is clearly reflected in the overlap function and the mean firing rate (see Fig. 2), and relatively large-amplitude oscillations in these order parameters with the same frequency as the sinusoidal weak input signal appear. However, by increasing further the level of noise *D*, we observe that the correlation with the input signal is lost. For a further increase of noise around a given value *D*_2_ (which in the simulations performed in the figure is about 265*pA*), a second peak in *C*(*D*) appears, where a strong correlation of the neural activity with the input weak signal is recovered. This noise level corresponds to the critical value of noise at which a second order phase transition between the oscillatory phase and a disordered phase emerges.

**Fig 4:**
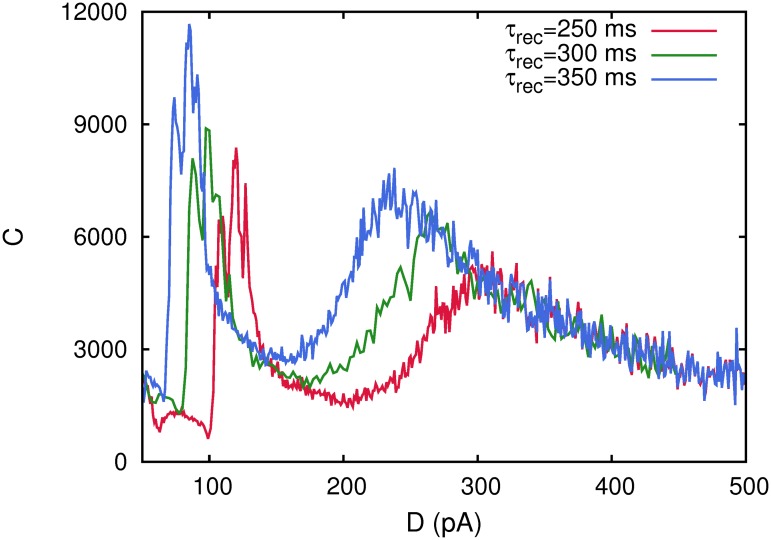
The behavior of the correlation *C*(*D*) for a single realization of a network of *N* = 2000 neurons with different levels of synaptic depression at the synapses monitored by varying *τ*_*rec*_. The figure shows that an increase of *τ*_*rec*_ causes the maxima of *C*(*D*) to appear at lower noise intensities. Other parameters were as in Fig. 1.

To check the influence of short term depression in the appearance of these maxima of the correlation function *C*(*D*), we have varied the neurotransmitter recovery time constant *τ*_*rec*_, which is a well know parameter that permits the tuning of the level of depression at the synapses. In fact, large recovering time constants are associated to stronger synaptic depression because the synapses need more time to have available neurotransmitter vesicles in the ready releasable pool. Therefore, we repeat the numerical study for several values of the time recovery constant *τ*_*rec*_ = 250, 300, 350 *ms*, considering a network of *N* = 2000 neurons. The results are depicted in Fig. 4, where *C*(*D*) is shown for different values of *τ*_*rec*_. Two main intriguing effects are observed. First, the maxima of the *C*(*D*), at which there is a high correlation with the weak signal, appear at lower noise intensities when *τ*_*rec*_ is increased. This is due to the fact that, when the level of synaptic depression is increased, the transitions between ordered and oscillatory phases and between oscillatory and disordered phases appear at lower values of noise intensity. This is due to the extra destabilizing effect over the memory attractors consequence of synaptic depression [20] and to the fact the maxima of *C*(*D*) occur precisely at these transitions points [17]. The second effect is that the correlation with the weak signal (the height of the maxima) increases with the level of depression, that is, the weak signal is processed with less noise, which is consequence of the phase transitions points—and therefore the maxima of *C*(*D*)—appear at lower noise values when synaptic depression is increased.

### The effect of short-term facilitation

**Fig 5:**
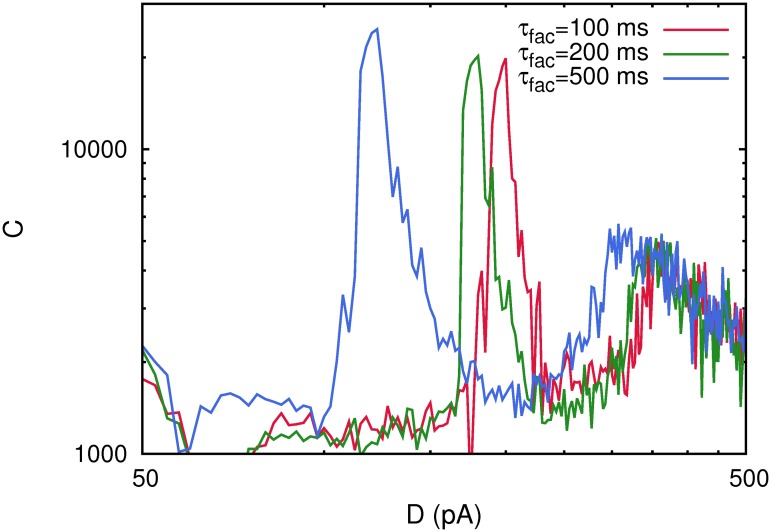
The correlation *C*(*D*) obtained for a single realization of the system with *N* = 800 neurons considering both short-term depression and short-term facilitation processes at the synapses with 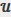 = 0.02. The figure illustrates that both maxima of *C*(*D*) appear at lower noise intensities as well as that the amplitude of the first maximum grows when *τ*_*fac*_ is increased. Other parameters were as in Fig. 1.

In general, synapses in the brain and, in particular, in the cortex can present—in addition to synaptic depression—the so called synaptic facilitation mechanism, that is an enhancement of the postsynaptic response at short time scales [18, 21]. Both *opposite* mechanisms can interact at the same time scale during the synaptic transmission in a complex way whose computational implications still are far of being well understood. The study of the influence of both mechanisms during the processing of weak signals in a neural medium, constitutes a very suitable framework to investigate this interplay. With this motivation, we present in this section a computational study of how synaptic facilitation competing with synaptic depression influences the detection of weak stimuli in a network of spiking neurons. In the following computational study, we consider a fixed time recovery constant *τ*_*rec*_ = 300 *ms*, which is within the physiological range of the actual value measured in cortical neurons with depressing synapses [21]. Also, we take several values for the characteristic facilitation time constant, namely, *τ*_*fac*_ = 100, 200, 500 *ms*, and 𝒰 = 0.02. The results obtained for the correlation function *C*(*D*) for a network of *N* = 800 neurons are depicted in Fig. 5. In this figure, we can observe a clear dependence between the level of noise at which maxima in the correlation function appear and the characteristic facilitation time constant *τ*_*fac*_. In particular, the figure shows that, as *τ*_*fac*_ increases, the maxima of *C*(*D*) emerge at lower noise intensities. Moreover, one observes that the intensity of the correlation at its low noise maximum grows whenever *τ*_*fac*_ is increased.

A possible explanation for this phenomenology is the following: it is well known, that facilitation favors to reach the stored attractors and their subsequent destabilization, in auto-associative neural networks [22]. In other words, synaptic facilitation favors the appearance of the oscillatory phase. So then, for the same level of noise *D*, more facilitated synapses induce an easy recovery and posterior destabilization of the attractors, and therefore, an easy transition to the oscillatory phase from the memory phase. This, in practice, means that the transition point between the two phases, appears at lower values of the noise for more facilitated synapses, and it is precisely at this transition point, where the low noise maximum of *C*(*D*) appears. On the other hand, synaptic facilitation favors the recovery of the memory attractors with less error [22]. In this way, when the transition to the oscillatory phase occurs, attractors are periodically and transiently recovered with less error during some time so that, the coherence of the activity of the system with the weak signals is larger since it is not affected by this extra source of noise. These findings provide a simple mechanism to control the processing of relevant information by changing the level of facilitation in the system which can be done, for instance, controlling the level of calcium influx into the neuron or by the use of calcium buffers inside the cells.

### The effect of network size

**Fig 6:**
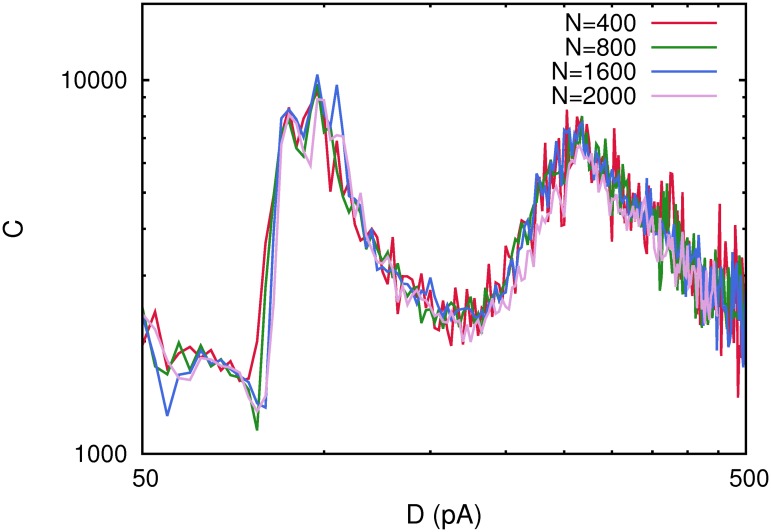
The correlation *C*(*D*) for different number of neurons in the network. The figure shows the same maxima and shape for *C*(*D*) independently of *N* which confirms that the results obtained are independent from the size of the network considered. Each curve has been obtained with a single realization of the corresponding network. Other parameters were as in Fig. 1.

In order to verify the robustness of the results reported above and to observe the possible effects that may arise due to the finite size of the system used in our simulations, we have carried out a study of the system increasing the number of neurons in the network as *N* = 400, 800, 1600, 2000, but maintaining the rest of the parameters and considering spiking neurons with pure depressing synapses. The computed correlation *C*(*D*) for all these cases is depicted in Fig. 6, which reveals that the main findings of our previous study remain and are independent of the number of neurons in the system. In fact, different *C*(*D*) curves for different values of *N* do not present significantly changes neither in their shape and intensity, nor in the level of noise at which the different maxima of *C*(*D*) appear. These results permit us to hypothesize that our main findings here are enough general and could also appear in large populations of neurons as in cortical slices or even in some brain areas.

### The effect of storing many patterns in the network

**Fig 7:**
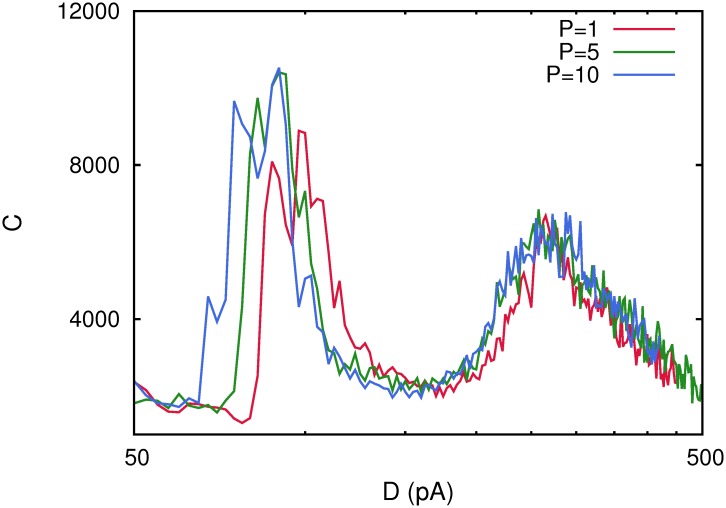
Correlation function *C*(*D*) for different number of stored patterns *P* in a network with *N* = 2000 neurons. This shows that as *P* increases the low-noise maximum of the correlation *C*(*D*) increases in intensity and appears at lower level of noise while the second correlation maximum remains unchanged. Each curve has been obtained with a single realization of the corresponding network. Other parameters values were as in Fig. 1.

In the studies reported in the above sections, we have considered just one activity pattern of information stored in the maximal synaptic conductances. We study now, how robust are these findings when the number *P* of activity patterns stored in the system increases with all other parameters of the model unchanged. In our study, we have varied *P* from 1 to 10 in a network of *N* = 2000 neurons with pure depressing synapses (*τ*_*rec*_ = 300 *ms*) and the corresponding correlation functions *C*(*D*) for all these cases are depicted in Fig. 7. One can appreciate that the phenomenon remains when *P* is increased, and while the maximum of *C*(*D*) that appears at high noise—around *D*_2_ = 265*pA*—does not dramatically change with *P*, the number of stored patterns has a strong effect on the maximum of *C*(*D*) appearing at low noise. In fact, this maximum appears at lower level of noise and with more intensity as *P* is increased.

A possible explanation for this intriguing behavior can be understood as follows. It is well known that in Hopfield binary neural networks the increase of the number of stored patterns induces interference among the memory attractors and therefore constitutes an additional source of noise that tries to destabilize them [23]. This also occurs in our spiking network and, in the presence of dynamic synapses, this destabilizing effect results in an early appearance of the transition between the memory phase and the oscillatory phase and therefore, in the appearance of the first low-noise maximum of *C*(*D*). Then, at relatively low levels of ambient noise *D* the transition from below to the oscillatory phase will occur at lower values of *D* in a network that stores a larger number of patterns. At relatively large values of the ambient noise, however, the main destabilizing effect is due to the ambient underlying noise, and therefore, the effect of increasing *P*, although is also present, it is less determinant. On the other hand, the amplitude of the maximum of *C*(*D*) increases with *P* because, as explained above, the transition among memory and oscillatory phases occurs at low value of the ambient noise for *P* larger. Then, the thermal fluctuations in the memory phase and during the phase transition to the oscillatory phase are lower. In this way, during the oscillations starting at the transition point, the attractors are recovered transiently with less error which induces the coherence with the weak signal to be larger.

### The effect of the asymmetry of the stored pattern

The features of the pattern-antipattern oscillations which characterize the oscillatory phase in neural networks with dynamic synapses (including short-term facilitation and depression), are highly dependent on the particular symmetry in the number of active and silent neurons in the stored pattern. This is controlled by the parameter *a* (introduced in the definition of the synaptic weights (4)). In all the results reported in previous sections, we have considered *a* = 0.5, which causes an oscillatory phase characterized by a regime of symmetric oscillations between an activity state correlated with the stored pattern and another with the same level of activity correlated with the antipattern. If we consider *a* ≠ 0.5, an asymmetry will be induced in the mean network activity, that is, there will be an excess of 1’s over 0’s or vice versa during pattern-antipattern oscillations. In fact, oscillations occurs between an high activity (Up) state and a low activity (Down) state. Moreover, this asymmetry in the activity of the stored pattern can have a strong influence in the phase diagram of the system and can cause even that the oscillatory phase does not emerge. Since the phenomenology of interest here—namely the emergence of an network activity correlated with a weak subthreshold stimulus—is highly dependent on the transition points at which the system moves over different phases, it is reasonable to think that the parameter *a* will have a strong influence on it.

**Fig 8:**
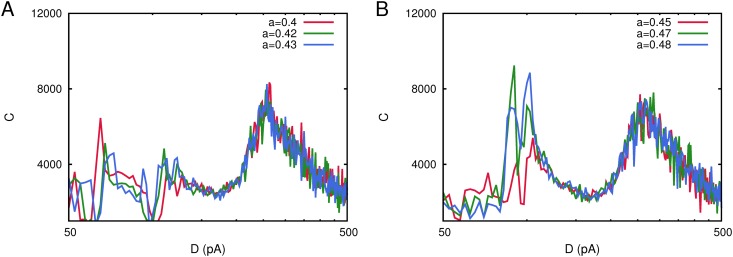
Changes in the shape of the correlation *C*(*D*) for a network with *N* = 800 neurons considering different levels of asymmetry in the network activity encoded in the stored pattern as measured with the parameter *a* (see main text for the explanation). Panel A depicts correlation curves for *a* ≤ 0.45, showing that the correlation *C*(*D*) drops around *D* = 100*pA* and another maximum starts to emerge at *D* = 65*pA*. Panel B illustrates *C*(*D*) curves for *a* ≥ 0.45, showing that the low noise maximum at *D* = 100*pA* looses intensity as *a* decreases. Each curve has been obtained with a single realization of the corresponding network. Other parameters were as in Fig. 1.

We have performed a computational study in a network of *N* = 800 neurons with pure depressing synapses (*τ*_*rec*_ = 300 *ms*) to investigate this particularly interesting issue, for which we have considered a single stored pattern *P* = 1 with *a* = 0.40, 0.42, 0.43, 0.45, 0.47, 0.48 and whose results are depicted in Fig. 8. We observe here a very interesting and intriguing effect in the shape of the correlation *C*(*D*) when *a* is varied. The maximum in the correlation between the network activity and the weak signal at low noise tends to disappear as the value of *a* decreases from the symmetric value *a* = 0.5. In fact, when *a* < 0.45 the correlation *C*(*D*) drops abruptly at that point, around *D*_1_ = 100*pA*. As we have explained above, a large level of asymmetry in the stored pattern could impede the appearance of the Up/Down transitions characteristic of the oscillatory phase which, therefore is absent. The consequence is that, there is not a transition point between a memory phase and an oscillatory phase which impedes the emergence of the low noise maximum of *C*(*D*). On the contrary, the second maximum of *C*(*D*), which appears at high noise around *D*_2_ = 265*pA*, remains invariant for all values of *a* studied here. The explanation to this second situation is also simple because, although asymmetry in the stored pattern is present, the phase diagram of the system still presents a phase of memory retrieval at low noise and a non-memory phase at large noise, separated by a second order phase transition point around which the second maximum of *C*(*D*) is originated.

### The effects of the underlying network topology

In previous sections, we have considered for simplicity—as our system under study—a fully connected network of spiking neurons. This is far to be the situation in actual neural systems, where neurons are not all connected to each others. In fact, biological neural systems are characterized by a underlying complex network topology which is consequence of different biophysical processes during their developing, including among others, exponential growth at early stages of developing and posterior synaptic pruning processes [24]. All these processes are also influenced by limitations in energy consumption in the system. In this section, we explore if the emergence of several maxima, as a function of the underlying noise, in the correlation between the network activity and some weak subthreshold stimulus, is altered when more realistic network topologies are considered.

**Fig 9:**
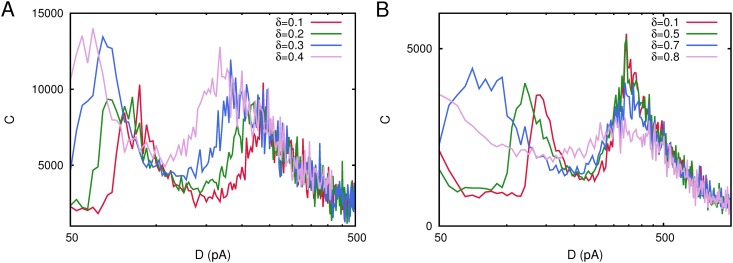
Behavior of the correlation *C*(*D*) for a diluted network. (A) As the fraction *δ* of removed connections increases both maxima of *C*(*D*) appear at lower noise intensity. The network size was set to *N* = 800 and a factor *N* instead of ⟨*k*⟩ is considered in the definition (4). Each curve has been obtained with a single realization of the corresponding network. Other parameters were as in Fig. 1. (B) In this case simulations has been performed considering diluted networks using the same procedure than in panel A, but with less level of synaptic depression, *τ*_*rec*_ = 200 *ms*, and synaptic weights normalized with a factor ⟨*k*⟩ = (1 − *δ*)^2^ *N*. Also in this case, the corresponding *C*(*D*) curves has been obtained for *N* = 200 and averaging over 10 different networks.

We have considered first the case of a random diluted network. We can configure this network topology starting, for instance, starting with a fully connected network and then removing randomly a certain fraction *δ* of the synaptic connections. In Fig. 9A, it is depicted the resulting correlation function *C*(*D*) for single realizations of diluted networks generated in this way with *N* = 800 and *δ* = 10%, 20%, 30% and 40%. The figure illustrates two main findings. First, the robustness of the main emergent phenomena described in the above sections also in this type of diluted networks, and second that, as the dilution grows and a higher fraction of connections is removed, both maxima of *C*(*D*) appear respectively at lower levels of noise. Moreover, if dilution is too high, it seems that the maximum appearing at low noise tends to disappear. This only can be consequence that the stable memory attractors lose stability—due to strong dilution—and disappear in the presence of ambient noise, in such a way that only an oscillatory phase and non-memory phases are present.

In our analysis with a diluted network, we performed dilution starting with a fully connected network where ⟨*k*⟩ = *N*. To avoid the possible effect of a factor 1/*N* normalizing the synaptic weights (4) during dilution, we have done an additional analysis considering a diluted network with a normalizing factor in the weights ⟨*k*⟩ = (1 − *δ*)^2^ *N*, which is the mean connectivity degree in the resulting diluted network with *δ* being the probability of a link to be removed during the dilution process. The corresponding results are summarized in the Fig. 9B. One can observe also that results are similar for this second type of dilution, that is, the low noise maximum of *C*(*D*) moves toward lower values of the ambient noise and even can disappear as dilution is increased. The main difference with the first type of dilutions is, however, that the level of noise at which the high noise maximum of *C*(*D*) is not dramatically affected by dilution. In Fig. 9B, the correlation curves *C*(*D*) have been obtained after averaging over 10 realizations of a network of *N* = 200 neurons with pure depressing synapses (with *τ*_*rec*_ = 200 *ms*).

**Fig 10:**
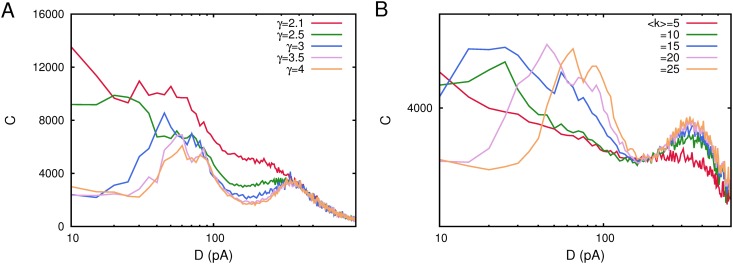
Detection of weak subthreshold stimulus as a function of noise *D* in spiking networks with complex scale-free topologies. (A) Correlation curves *C*(*D*) obtained for different values of the exponent *γ* of the degree connectivity distribution *p*(*k*) ∼ *k* ^−*γ*^ in a network with ⟨*k*⟩ = 20. The figure shows that around *γ* = 3 multiple maxima in *C*(*D*) start to emerge. (B) The same correlation curves *C*(*D*) for scale free networks with *p*(*k*) ∼ *k* ^−3^ and different values of the mean connectivity degree ⟨*k*⟩. Again for more realistic large values of ⟨*k*⟩ around 20 multiple maxima in *C*(*D*) start to appear. All curves have been obtained for *τ*_*rec*_ = 200 *ms* in a network with *N* = 200 neurons and after averaging over 10 different network realizations. Other parameters as in Fig. 1.

Diluted networks, however, are homogeneous and do not introduce complex features which could induce more intriguing behavior in the system. Even more interesting and realistic concerns the case of networks with complex topology such as the so called scale-free networks, where the node degree probability distribution is *p*(*k*) ∼ *k* ^−*γ*^, with *k* being the node degree. In fact, it has been recently reported that these complex topologies can induce additional correlation with the network activity and the processed weak stimulus due to the network structural heterogeneity [13]. Fig. 10 summarizes our main results concerning the case of complex networks with scale-free topology. Correlation curves *C*(*D*) have been obtained after averaging over 10 realizations of a scale-free network of *N* = 200 neurons with pure depressing synapses (with *τ*_*rec*_ = 200 *ms*) and all other model parameters as in Fig. 1. We see that similarly to the cases studied above, two maxima in the correlation function *C*(*D*) emerge—for two well defined values of the underlying noise—also in scale-free networks. However, we do not observe the emergence of an additional maximum of *C*(*D*) which could be induced only by the topology. As it has been reported in [13], this maximum should appear at low values of the ambient noise, for *γ* ∼ 3. The existence of a robust oscillatory phase when dynamic synapses are considered and a phase transition between memory and this oscillatory phase at relatively low values of the ambient noise could hidden the appearance of the this maximum due to the existence of a low noise maximum of *C*(*D*) around this phase transition. In any case, as it is depicted in Fig. 10, the emergence of two maxima in *C*(*D*) is a robust phenomenon also in these complex scale-free network topologies for a wide range of the relevant network parameters such as the exponent of the network degree distribution (Fig. 10A) and the mean connectivity in the network (Fig. 10B). Interestingly is that the low noise maximum seem to start to emerge for values of *γ* ≳ 3 and values of the mean connectivity ⟨*k*⟩ ≳ 15–20. These values corresponds to realistic ones, since, for instance, most of the actual complex networks in nature have degree distributions with *γ* between 2 and 3 [25]. Moreover neurons in the brain of mammals have large connectivity degrees and realistic values of the mean structural connectivity in cortical areas of mammals has been reported to be around ⟨*k*⟩ ≈ 20 [26].

**Fig 11:**
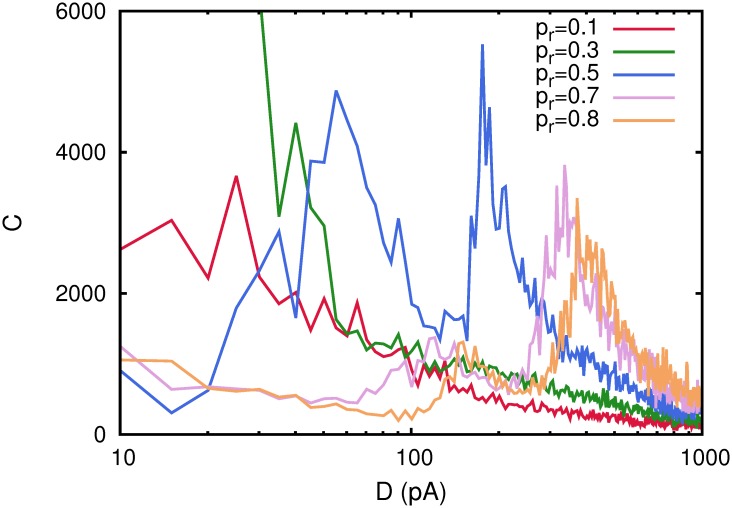
Detection of weak subthreshold stimulus as a function of noise *D* in a WS small-world network a function of the rewiring probability *p*_*r*_. The network has *N* = 200 neurons each one with *k*_0_ = ⟨*k*⟩ = 20 neighbors and depressing synapses with *τ*_*rec*_ = 100 *ms*. Different correlation functions *C*(*D*) have been obtained after averaging over 10 different network realizations. Other parameters as in Fig. 1.

Finally, we have consider in our study the case of a complex network with the small-world property. A prominent example of such type of networks is the so called Watts-Strogatz (WS) network [19]. These networks are generated starting with a regular network where each node, normally placed in a circle, has *k*_0_ neighbors. Then, with some probability *p*_*r*_, know as probability of rewiring, each link among nodes in this regular configuration is rewired to a randomly chosen node in the network avoiding self-connections and multiple links among two given nodes. In this way for *p*_*r*_ = 0, one has a regular network with *p*(*k*) = *δ*(*k* − *k*_0_) and for *p*_*r*_ = 1 one has a totally random network with *p*(*k*) being a Gaussian distribution centered around *k*_0_. Note that for varying *p*_*r*_ one always has ⟨*k*⟩ = *k*_0_. We have placed neurons defined by the dynamics (1) in such WS networks and studied as a function of the underlying noise the emergence of correlations between the network activity and some subthreshold weak signals by means *C*(*D*). The results are summarized in Fig. 11 where each *C*(*D*) curve has been obtained after averaging over 10 realizations of a network with *N* = 200 neurons and pure depressing synapses with *τ*_*rec*_ = 100 *ms*. One can see that the appearance of several maxima for *C*(*D*) occurs for values of *p*_*r*_ ≳ 0.5. More precisely, the low noise maximum does not emerge for low rewiring probabilities, which clearly indicates that the memory phase does not appear for such small values of *p*_*r*_ for the whole range of noise *D* considered here. Also this finding suggests the positive role of long range connections—that only can emerge with high probability when *p*_*r*_ is high—for the existence of such low noise maximum in *C*(*D*). In fact, the emergence of a memory phase can be only understood when in the network appear such long range connections since the stored memory patterns involve these type of spatial correlations among active and inactive neurons.

### Use of more realistic weak signals

**Fig 12:**
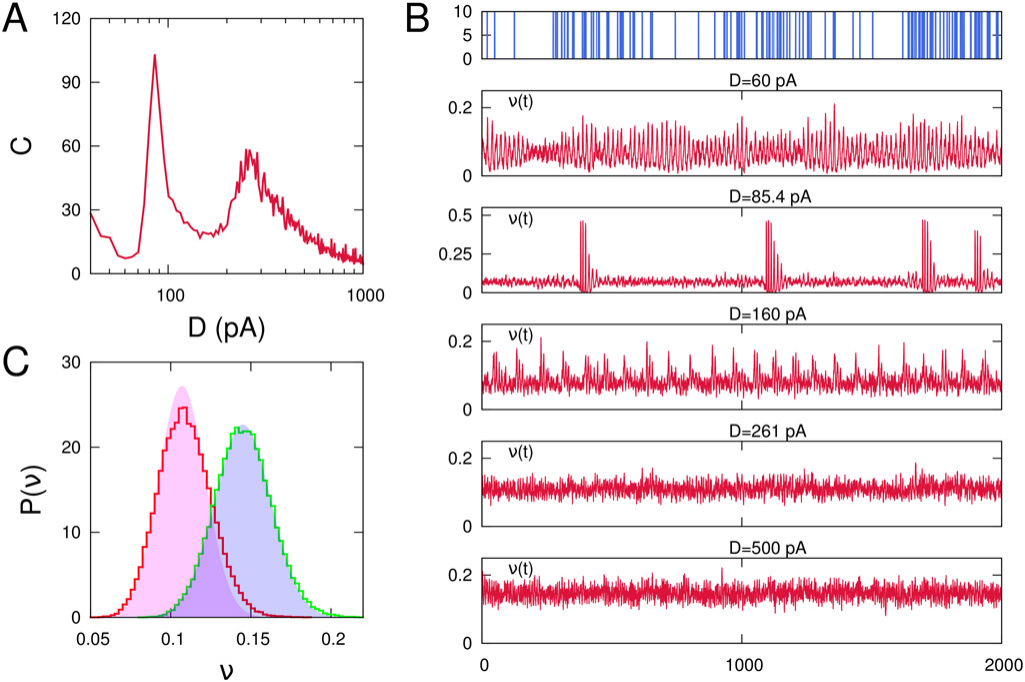
Detection of more realistic Poissonian weak stimuli as a function of the noise noise *D*. (A) Corresponding correlation *C*(*D*) for a network of *N* = 400 neurons depicting several maxima similarly to the case of sinusoidal stimuli. The curve has been obtained after averaging over 20 network realizations. The weak signal current $I_{i}^{ext}$ is the resulting of an inhomogeneous Poissonian train of spikes of amplitude *d*_*s*_ = 10*pA* injected in each neuron (see panel B top graph) at frequency *λ*(*t*) = *λ*_0_[1 + *a*sin(2*πf*_*s*_ *t*)] with *λ*_0_ = 70*Hz* and *a* = 0.75. (B) Time-series of the network mean firing rate for increasing values of the noise intensity *D* along the correlation curve depicted in panel A. (C) Normalized histograms of the network mean-firing rate at the high noise maximum of *C*(*D*) (red curve) corresponding to the critical point *D*_*c*_ = 261*pA* and for a higher level of noise, namely *D* = 500*pA*, above this critical point (green curve). The colored areas corresponds to the best Gaussian fitting of both histograms. Other parameters were as in Fig. 1.

In actual neural systems is expected that relevant signals arrive to a particular neuron in the form of a spike train, with relevant information probably encoded in the timing among the spikes. In this sense, the use of a sinusoidal weak current to explore how the system detect it in all cases considered above could not be the most realistic assumption (it could be enough realistic if relevant information will be encoded in subthreshold oscillations instead that in the precise timing of the spikes). To investigate the ability of the system to detect and process the with more realistic weak signals in the presence of noise we have considered the input weak signal as an inhomogeneous Poisson spike train with mean firing rate *λ*(*t*) = *λ*_0_[1 + *a*sin(2*πf*_*s*_ *t*)], being *λ*_0_, *a* positive constants. In this way, relevant information is encoded as a sinusoidal modulation of the arrival times of the spikes in the train. Fig. 12A depicts the coherence among mean firing rate in the network and this weak signal (which is shown in the top graph of Fig. 12B). The correlation curve *C*(*D*) has been obtained after averaging over 20 realizations of a fully connected network with *N* = 400 neurons and pure depressing synapses with *τ*_*rec*_ = 300 *ms*. The figure clearly illustrates that also in this more realistic case the system present a strong correlation with the weak signal at different levels of noise at which phase transitions among different non-equilibrium phases appear (see time series for increasing level of noise from top to bottom in Fig. 12B). These are a memory phase where active neurons in the stored memory pattern are strongly synchronized (*D* = 60*pA*) (population burst regime), a transition point characterized by signal driven high-activity (Up state)/low-activity (Down state) oscillations (*D*_1_ = 85.4*pA*), a phase of intrinsic Up/down oscillations (*D* = 160*pA*), a critical point toward a non-memory phase characterized by signal driven fluctuations plus thermal fluctuations (at *D*_*c*_ = *D*_2_ = 261*pA*), and a non-memory phase, or asynchronous phase, characterized by constant firing rate with Gaussian thermal fluctuations (for instance at *D* = 500*pA*). In fact, in Fig. 12C it is depicted the difference in steady state features of the behavior for the two last cases. That is, at the critical point *D*_2_ the stationary distribution of the resulting mean firing rate has a bias toward positive fluctuations induced at the exact arrival time of the weak signal spikes. This as evidenced by the disagreement between this distribution (red curve in Fig. 12C) and the shaded red area, which represents the best fit to a Gaussian distribution. On the other hand, during the asynchronous state for *D* = 500*pA* the same steady state distribution (green line in Fig. 12C) is clearly Gaussian (shaded blue area) without presenting a bias with the timing of signal spikes.

## Discussion

We investigated in great detail by computer simulations the processing of weak subthreshold stimuli in a auto-associative network of spiking neurons—*N* integrate and fire neurons connected to each others by dynamic synapses using different network configurations—competing with a background of ambient noise. In particular, we studied the role of short-term synaptic depression in the efficient detection of weak periodic signals by the system as a function of the noise. Our results show the appearance of several well defined levels of noise at which there is a strong correlation between the mean activity in the network and the weak signal. More precisely, in the range of noise intensities considered in this study, the transmission of the information encoded in the weak input signal to the network activity is maximum when noise intensity reaches two certain values.

The maximum or peak appearing at relatively low levels of ambient noise *D*_1_, corresponds to a transition point where the activity of the network switches from a memory phase—in which a stored memory pattern is retrieved—to an oscillatory phase where the system alternatively is recalling the stored pattern and its anti-pattern in a given aperiodic sequence. Thus, at this level of noise and in the presence of the weak signal this oscillatory behavior of the network activity becomes correlated with the signal oscillating at its characteristic frequency. This fact provides an efficient mechanism for processing of relevant information encoded in weak stimuli in the system since at this transition point, for instance, the system could efficiently recall different sequences of patterns of information according to predefined input signals.

On the other hand, the second maximum which appears at relatively high level of ambient noise, namely *D*_2_, emerges around a second order phase transition between the oscillatory phase explained above and a disordered or non-memory phase, where the system is not able to recall any information stored in the patterns. Although the resulting network activity around this maximum is highly noisy, it is strongly correlated with the weak signal, appearing a modulation of the noisy activity that follows the signal features (see the case *D*_2_ = 265*pA* in Fig. 2).

We also studied in detail, the influence that the particular level of synaptic depression could have in the appearance of the two maxima in the correlation between the network activity and the weak stimulus. By changing the recovery time constant of the active neurotransmitters, for instance, we observe that the longer the synapses take to recover the neurotransmitters (larger *τ*_*rec*_), the lower level of ambient noise is needed to induce to reach the different maxima of the corresponding correlation function (see the Fig. 4).

Furthermore, we have observed that short-term facilitation competing with short-term depression at the synapses induces also additional intriguing effects in the way system responds to the weak stimulus in the presence of noise. Our results reveal, that for larger values of the characteristic time constant characterizing the facilitation mechanism, namely *τ*_*fac*_, the maxima in the correlation *C*(*D*) between the network activity and the weak signal appear at lower levels of ambient noise than in the case that only synaptic depression is considered. In addition, we can observe that the correlation at the low noise maximum amplifies when the level of synaptic facilitation increases (see Fig. 5). Both phenomena can be understood taking into account that facilitation favors the retrieval of information in the attractors and their posterior destabilization, which is the origin of the oscillatory phase. Then, an increase of facilitation moves the transition point between the memory phase and the oscillatory phase towards lower values of the ambient noise *D*. Also, facilitation favors the recovery of the memory attractors with less error, which implies that, when the coherence with the weak signals emerges in the network activity, it is affected by less sources of noise, and therefore, it increases for large facilitation.

We proof as well the robustness of the results reported here, by checking that the appearance of several maxima in the correlation *C*(*D*) between the network activity and weak subthreshold stimuli, around some phase transitions points remains for larger number neurons *N* or number of stored patterns *P*. Our study reveals that our main findings are independent of the network size (see Fig. 6) and therefore could probably also obtained in actual neural media. Nevertheless, when we study the correlation *C*(*D*) for different number of stored patterns, some new effects appear. In fact, the low noise maximum of *C*(*D*) tends to disappear when the number of stored patterns is increased. The reason is, that an increase in *P* induces the appearance of the oscillatory phase at lower values of the ambient noise *D* and, consequently, the development of this maximum of *C*(*D*) occurs at lower noise (see Fig. 7). On the other hand, the appearance of the second high noise maximum of *C*(*D*) is not significantly affected by an increase of *P* (see also Fig. 7).

Another interesting result is obtained when we consider an asymmetric stored memory pattern, that is, *a* ≠ 0.5, so that there is an excess of 1’s over 0’s in the stored pattern or vice versa. In Fig. 8, we can see that by increasing the pattern asymmetry (reducing *a*), the low noise maximum of *C*(*D*) decreases. This is mainly due to the fact that oscillations between the high-activity and low activity states, characterizing the oscillatory phase, tend to be less visible or even disappear—the network activity is quasi clamped in the memory attractor—for more asymmetric stored patterns. In fact, if we consider *a* < 0.45, the low noise maximum of *C*(*D*) drops abruptly and the system is not able anymore to process the information encoded in the weak signal at this level of ambient noise (see Fig. 8).

We have performed also a complete study of how our main findings are influenced by the given network topology. As a first step in this research line, we have considered the case of diluted networks. We built different types of diluted networks by erasing a fraction of links at random in a fully-connected network. In all cases that we have considered, the main results still emerge, that is the existence of several maxima in the correlation *C*(*D*) between the network activity and the weak stimulus at some precise levels of noise around non-equilibrium phase transitions. If the fraction of erased synaptic connections is larger, these maxima of *C*(*C*) appear at lower level of ambient noise. Moreover, there is a tendency that leads to the complete disappearance of the low noise maximum for strong dilution (see Fig. 9). These findings are due to the fact that, dilution of synaptic links diminishes the memorization and recall abilities of the network (since memory pattern features are stored in these links). The consequence is that, in more diluted networks, the memory phase appears at lower values of ambient noise, and can even disappear in absence of noise for very diluted networks. Secondly, we have consider also the case of complex networks with scale-free properties in the degree distribution and with the small-world property. In all cases there is a wide range of the relevant networks parameters, such as the exponent of the scale-free degree distribution, the mean connectivity in the network and the rewiring probability in the the case of the WS small world network, for which *C*(*D*) shows also similar maxima at given noise levels where the system efficiently process the weak stimulus. Moreover this range of parameters is consistent with those measured in actual neural systems.

Note that the consideration here of some complex networks with scale-free topology, introduces node-degree heterogeneity in the system. This fact induces also neuron heterogeneity in the sense that more connected neurons can be more excitable than less connected neurons, so one can have different levels of neuron excitability in the system. However, we have seen that even in this case there is a wide range of model parameters where the relevant phenomenology here still emerges. This tell us that in a more general scenario where different types of neuron heterogeneity are considered, the phenomena reported here also will emerge.

After our analysis in the present work, we conclude that the efficient detection or processing of weak subthreshold stimuli by a neural system in the presence of noise can occur at different levels of this noise intensity. This fact seems to be related to the existence of phase transitions in the system precisely at this levels of noise, a suspicion which is presently been analyzed in greater detail. In the cases studied here, within the range of noise considered, there is a maximum in the correlation *C*(*D*) of the network activity with the stimulus which seems to correspond to a discontinuous phase transition (the maximum at relatively low ambient noise) as well as another maximum appearing around a continuous phase transition (the maximum at relatively high noise). The difference in the type of the emerging phase transition determines the way the weak subthreshold stimulus is processed by the neural medium.

We hope to study next the computational implications that each one of theses maxima induces and their possible relation with high level brain functions. In particular, in some preliminary simulations reported in the present work with more realistic Poissonian signals, a detailed inspection of the network activity temporal behavior, compared with the weak signal time series around the low noise maximum, show a strong resemblance with working memory tasks, where relevant information encoded in the input signal is maintained in the network activity during some time, even when the input signal has disappeared. Also in the case of several memory pattern stored in the system and around this low noise maximum, the system could process a given sequence of patterns encoded in the stimulus. Precisely at this level of noise, a non-equilibrium phase emerges characterized by continuous sequence of jumps of the network activity between different memories, which could be correlated with a particular sequence of memories in the presence of an appropriate stimulus. On the other hand, at the second resonance peak is the precise timing between input spikes which are detected and processed by the network activity.

**Fig 13:**
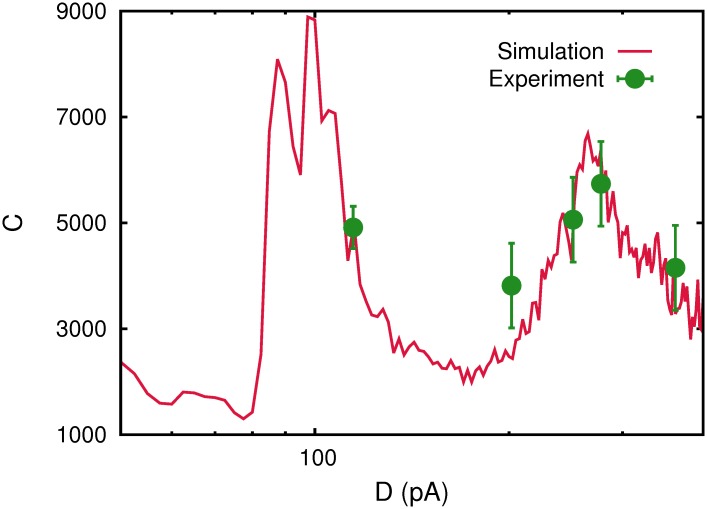
Fitting experimental data in the auditory cortex to our model. The experimental data (symbols with the corresponding error bars) reported in [8] are compared as a function of noise with the correlation function *C*(*D*) described in Fig. 1 corresponding to a single realization of a spiking network of *N* = 2000 integrate and fire neurons (red solid line) and dynamic synapses. The experimental data *C* (in arbitrary units) has been multiplied by a factor 10^4^, and the noise amplitude *M* (in *dB*) has been transformed in our noise parameter *D* using the nonlinear relationship $D=D_{0}+\frac{M\eta }{2}\left\{1+\text{erf}\left[(M-M_{0})/\sqrt{2}\sigma_{M}\right]\right\}$ with *D*_0_ = 0.1*pA*, *η* = 0.71(*pA*/*dB*), *M*_0_ = 50*dB* and *σ*_*M*_ = 140*dB*.

Finally, we mention that it would be interesting to investigate if the relevant phenomenology reported in this work could emerge naturally in actual systems. In fact, recent data from a psycho-technical experiment in the human brain [8] can be better interpreted, using different theoretical approaches and dynamic synapses, considering the existence of several levels of noise at which relevant information can be processed [17, 27]. In Fig. 13 it is shown how these experimental data can be also interpreted in terms of the correlation function *C*(*D*) obtained within the more realistic model approach reported in this paper, that is, a complex network of spiking neurons. This should serve as motivation to study in depth how neural systems process weak subthreshold stimuli in a more biological and realistic scenario. For instance, one could consider conductance based neuron models instead of the simplified integrate and fire model used here or conceive more realistic stimuli, and other complex network topologies. The last could include, for instance, different type of node degree-degree correlations [28–30] or network-network correlations constituting a *multiplex* structure as a recent work suggests to occur in the brain [31]. All these additional considerations could provide some new insights in order to design a possible experiment easily reproducible by biologists to investigate the emergence the phenomena reported here in actual neural systems. On the other hand, the relation of the relevant phenomenology reported in this study with the existence of different phase transitions in our system could be of interest for neuroscientists to investigate the existence of phase transitions in the brain.

## Supporting Information

S1 Parameter Values (XLS)

S2 Raw Data (TAR)
