# Supplementary figures and images for: Efficient Transmission of Subthreshold Signals in Complex Networks of Spiking Neurons
Source: PLoS One. 2015 Mar 23;10(3):e0121156. doi: 10.1371/journal.pone.0121156 (PMC4409401; doi:10.1371/journal.pone.0121156)

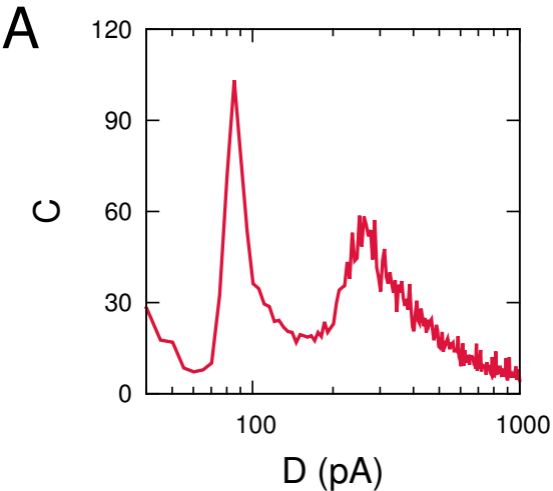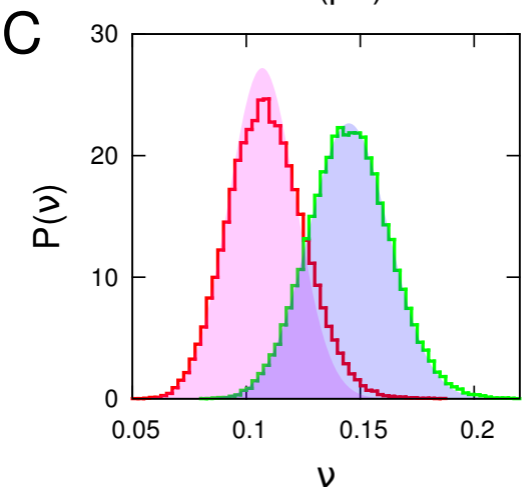

Supplement: S2 Raw Data — (TAR) [file pone.0121156.s002.tar › Fig12A_C.pdf]
